# Mild Fractionation of Hydrophilic and Hydrophobic Components From *Neochloris oleoabundans* Using Ionic Liquids

**DOI:** 10.3389/fbioe.2019.00284

**Published:** 2019-10-25

**Authors:** Rupali K. Desai, Maria Salvador Fernandez, Rene H. Wijffels, Michel H. M. Eppink

**Affiliations:** ^1^Bioprocess Engineering, AlgaePARC, Wageningen University, Wageningen, Netherlands; ^2^Faculty of Biosciences and Aquaculture, Nord University, Bodø, Norway

**Keywords:** biorefinery, hydrophobic compounds, hydrophilic compounds, ionic liquids, microalgae

## Abstract

Microalgae are a promising source for proteins, lipids, and carbohydrates for the food/feed and biofuel industry. To make microalgae production economically feasible, it is necessary to optimally use all produced compounds keeping full functionality. Therefore, biorefining of microalgae is the key to lower the cost of algal products using mild and effective processing techniques. In this article, we have tested the feasibility of aqueous solutions of imidazolium and phosponium ionic liquids to selectively milk the hydrophobic lipids from *Neochloris oleoabundans* biomass out of intact cells and recover after cell disruption the hydrophilic fraction containing proteins and carbohydrates. The results showed that the ionic liquid tributylmethylphosphonium methylsulfate (TBP SO_4_; Cyphos 108) is able to permeabilize fresh intact cells of *N. oleoabundans* for extracting 68% of total lipids out of the cells, whereas, after cell disruption, 80% of total proteins, and 77% of total carbohydrates could be obtained in aqueous buffers. This concept kept the recovered proteins in their native form without interacting with the ionic liquids that will denature the proteins. Selective biorefinery of different components from microalgae using ionic liquid TBP SO_4_ explains the novelty of this concept.

## Introduction

Microalgae are promising feedstocks for biofuel production. These photosynthetic microorganisms have high lipid productivity and do not compete for arable land when compared to terrestrial oleaginous crops (Wijffels and Barbosa, 2010). Microalgae have a very tough cell wall and thus require energy-intensive unit operations to break open the cell and release the intracellular content. Thus, despite the high lipid productivity, the energy input to separate the lipids is much higher than the energy obtained from the biomass, indicating the necessity to use less energy-intensive unit operations. Apart from lipids, microalgae are also good sources of proteins, carbohydrates, and pigments. Utilization of these value-added co-products for food, cosmetics, health, and chemicals would help in making the process economically feasible (Vanthoor-Koopmans et al., 2012).

The current process focuses on recovery of a single component from microalgae, i.e., lipids for biodiesel production (Cuellar-Bermudez et al., 2015). Most commonly, organic solvents are used for extraction of lipids. The Soxhlet (1879) method uses hexane as a solvent and the Bligh and Dyer's (1959) method uses chloroform and methanol mixture as a solvent for extraction. As these processes are designed to extract one component (lipids), it degrades the biomass, making it unsuitable for recovering other components (e.g., proteins, carbohydrates). Additionally, lipids can also be extracted using sub- and super-critical fluids (Herrero et al., 2006); however, these methods have high energy requirements and thus impact the overall economics of the process. In a study done by Ursu et al. (2014), protein extraction was performed using alkaline condition. However, the proteins precipitate and hydrolyzes under alkaline condition and had lower functional properties (Ursu et al., 2014). It is thus prudent to develop a mild process to fractionate the biomass into its components such that their value and functional integrity are retained.

Conventional extraction processes based on volatile organic solvents pose safety concerns, are toxic, and denature proteins. It is thus necessary to develop newer methods to address these issues. Some of the newer methods include use of supercritical fluids and, recently, ionic liquids (ILs).

ILs are salts that are liquid at temperatures below 100°C. They are composed of cations and anions and have negligible vapor pressure. They are known as designer solvents as their properties such as polarity and viscosity can be tailored by using a different combination of cation and anion (Freemantle, 1998). This makes IL an attractive solvent for liquid–liquid extraction.

ILs were used for lipid extraction from microalgae at elevated temperatures and together with co-solvents such as methanol (Young et al., 2010; Kim et al., 2012). Studies using mixtures of ILs have also been performed to extract lipids from algae biomass (Choi et al., 2014; Yu et al., 2015) and dissolution of microalgae in ILs were also demonstrated (Fujita et al., 2013). Teixeira in his studies have shown energy-efficient deconstruction of algae biomass by dissolution and hydrolysis of microalgae in ILs at temperatures above 100°C (Teixeira, 2012). Olkiewicz et al. (2015) showed ~75% lipid and 93% FAMEs recovery using hydrated phosphonium IL under ambient temperature conditions. All these studies together with some recent investigations (Orr and Rehmann, 2016; Orr et al., 2016; Wahidin et al., 2016; To et al., 2018) have established the potential of ILs to extract lipids from microalgae with high efficiency. While both Teixeira (2012) and Olkiewicz et al. (2015) have qualitatively demonstrated that all components of microalgae (lipids, proteins, and carbohydrates) can be recovered in one process after hydrolyzing the microalgae, it does not give any indication about recovery of proteins and carbohydrates and the stability of the more fragile proteins. Most of these studies address the recovery and extraction efficiency of lipids from microalgae showing a single-component isolation strategy. While recovery of other components such as the high-value proteins and carbohydrates are not addressed or that such harsh conditions are used (Wang and Zhang, 2012; Lee et al., 2017; To et al., 2018), the products are degraded/denatured. A recent study by Yu et al. (2015) reported energy-efficient extraction of lipids from *Chlorella vulgaris* using IL combined with CO_2_ capture. The study showed ~75% lipid (~89% FAMEs) recovery but the proteins were denatured in the process. Therefore, to be able to recover all components in their full functional state from microalgae biomass, it is necessary to use mild techniques. Majority of the articles discussed above are focused on lipid extraction from microalgae with ILs using harsh methods such as high temperature (100°C) that degrades the more fragile proteins and not biorefining all the functional biomass components. There is thus need to develop a process that is mild, i.e., does not degrade the proteins and thus helps recovering all components.

**Figure 1:**
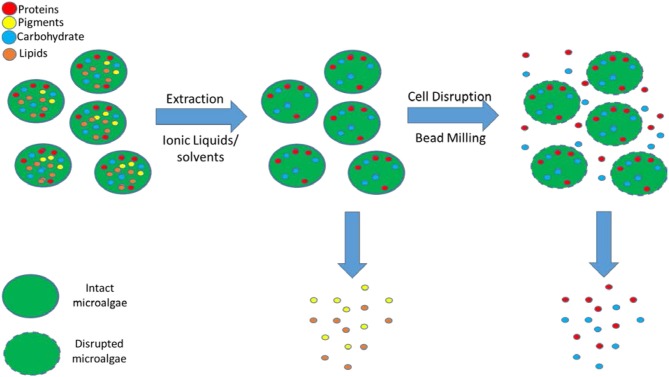
Extraction of the hydrophobic compounds (lipids, pigments) after pre-treatment with ILs/solvents followed by cell disruption with bead milling for the recovery of hydrophilic (proteins, carbohydrates) compounds.

The primary objective of this article is to develop a novel mild biorefinery concept whereby the algal biomass is fractionated into a hydrophobic fraction (lipids) by milking the lipids out of intact cells using mild pre-treatment at low temperature with an aqueous solution of IL followed by cell disruption with bead milling to obtain the more fragile hydrophilic fraction (proteins, carbohydrates) in their functional state with aqueous buffer solutions (Figure 1).

This study is in-line with a recent study we performed by developing a technology able to separate the hydrophilic (e.g., proteins, carbohydrates) and hydrophobic (e.g., pigments) components in their functional state after complete cell disruption with bead milling using emulsion-based IL separations (Desai et al., 2018). The microalgae strain used in this study is *Neochloris oleoabundans*, which is a high lipid- and protein-producing strain (Gouveia et al., 2009). Both fresh and freeze-dried algae were studied to understand the influence of IL pre-treatment on extraction efficiency of individual components and the stability of proteins.

## Materials and Methods

### Description of the Materials

The ILs used in this study were ≥95% pure and used without further purification. All the ILs listed in Table 1 were purchased from Iolitec. Chemicals and organic solvents used in the study, ethyl acetate, hexane, chloroform, methanol, sulfuric acid, phenol, and fatty acid standards were bought from Sigma.

**Table 1:** Ionic liquids used in the study.

**Ionic liquid names**		**Abbreviations**
1	Tributylmethylphosphonium methylsulfate (Cyphos 108) >95%	TBP SO_4_
2	Triisobutylmethylphosphonium tosylate (Cyphos 106) >95%	TBP TOS
3	1-Butyl-3-methylimidazolium dibutylphosphate 97%	BMIM DBP
4	1-Ethyl-3-methylimidazolium dibutylphosphate 97%	EMIM DBP
5	1-Butyl-3-methylimidazolium acetate >98%	BMIM acetate
6	1-Butyl-3-methylimidazolium dicyanamide >98%	BMIM DCA

### Microalgae Cultivation

*Neochloris oleoabundans* was cultivated in the laboratory in fresh water medium as described by Breuer et al. (2012) and the algae were stressed to have a higher lipid content. The microalgae were then harvested by centrifugation (4,000 rpm for 10 min). The microalgae were freeze-dried and used for extraction studies. For the study, using fresh cells, the algae were grown at the AlgaeParc pilot facility, Wageningen, The Netherlands. The cell suspension was centrifuged at 4,000 rpm for 10 min and used for the study.

### Pre-treatment With ILs and Fractionation of Biomass

**Figure 2:**
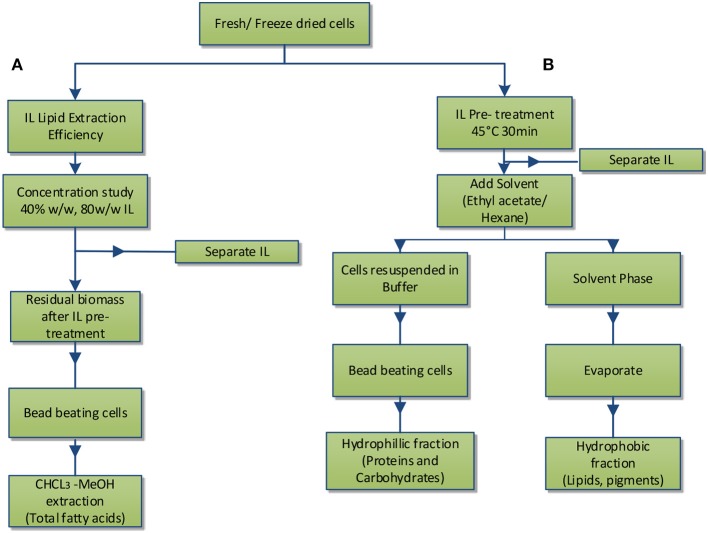
Experimental scheme: **(A)** IL lipid extraction efficiency; **(B)** IL pre-treatment and separation of hydrophobic and hydrophilic components.

As shown in Figure 2, two studies were performed, lipid extraction efficiency of IL from intact microalgae cells at two different IL concentrations (A) and IL pre-treatment of microalgae followed by subsequent fractionation into hydrophilic and hydrophobic components (B).

*Neochloris oleoabundans* cells (~10 mg) (freeze dried and/or fresh cells) were treated with 1.5 ml of aqueous solution of IL (see Table 1) at 45°C for 30 min. Fresh and freeze-dried cells used in the study were from different batches.

In study A, preliminary screening studies on IL pre-treatment of microalgae were conducted using the ILs in Table 1. The cells were pre-treated with 1.5 ml of 40% aqueous solution of IL at 45°C for 30 min followed by centrifugation at 3,000 rpm for 10 min removing the IL and then contacted with 3 ml of solvent (ethyl acetate) for 2 h. The hydrophobic components from microalgae (lipids) were extracted in the solvent phase and the amount of lipids extracted in the IL phase was determined by measuring the residual amount of lipids remaining in the cells (section Fatty Acid Determination). After the preliminary screening studies, TBP SO_4_ and BMIM DBP were selected and further studied, whereby the cells were pre-treated with 1.5 ml of 40 and 80% aqueous solution of IL at 45°C for 30 min. The cells, after pre-treatment, were separated from ILs by centrifugation at 3,000 rpm for 10 min and then contacted with 3 ml of solvent (ethyl acetate) for 2 h. The hydrophobic components from microalgae (lipids) were extracted in the solvent phase and lipid content was determined.

In the B study, the cells were pre-treated with 1.5 ml of 40% aqueous solution of BMIM DBP and TBP SO_4_ at 45°C for 30 min. The cells, after pre-treatment, were separated from ILs by centrifugation at 3,000 rpm for 10 min and then contacted with 3 ml of solvent (ethyl acetate) for 2 h. The hydrophobic components from microalgae (lipids) were extracted in the solvent phase and lipid content was determined. The microalgae cells remaining after pre-treatment, containing the hydrophilic components mainly proteins and carbohydrates, were suspended in buffer, beaten, and finalized by analyzing the protein (section Protein Content) and carbohydrate (section Carbohydrate Analysis) content.

### Fatty Acid Determination

The total fatty acids (FA) present in the microalgae were determined by treating the cells with CHCl_3_-MeOH as described by Breuer et al. (2013). For concentration studies, the total amount of fatty acids extracted in the IL phase was determined by measuring the residual amount of fatty acids remaining in the cells after pre-treatment with IL and is expressed as follows:

(1) $$\begin{array}{l} \%\text{Total FA extracted in IL per mg of biomass}\\ \;=\text{\%Total FA in control sample}\\ \;-\%\text{Total residual FA in the cells after pretreatment} \end{array}$$

For calculating the total FA content of IL and solvent-treated cells, the solvent phase (ethyl acetate/hexane) was evaporated under N_2_ stream and the residue was analyzed for total FA content after transesterification. The samples were measured in duplicate. The samples were analyzed in the GC (Agilent 7890A) and the run time was 30 min. For control sample, the fatty acid content was determined by treating the cells with CHCl_3_-MeOH as described by Breuer et al. (2013).

### Protein Content

Protein content was determined with a commercial assay kit (DC™ Protein assay, Bio-Rad, U.S.) using bovine serum albumin (Sigma–Aldrich A7030) as protein standard. The microplate assay protocol was used and the absorbance was measured at 750 nm using a microplate reader (Infinite M200, Switzerland). The cells after pre-treatment with IL were suspended in 1 ml of lysis buffer, 60 mM Tris, and 2% SDS, pH 9, in lysing matrix D tubes (6,913–500, MP Biomedicals Europe). The sample was bead beated for 3 cycles of 60 s at 6,500 rpm with a pause of 120 s between each cycle (Precellys 24, Bertin Technologies). The cell suspension was than heated at 100°C for 30 min. The cell suspension was separated by centrifugation and the supernatant was analyzed for protein content using the DC™ Protein assay. To determine the total protein content (control sample), the cells were directly resuspended in the lysis buffer, and analyzed as described above without any pre-treatment of cells. The samples were measured in duplicate. The protein is expressed as the % of total protein in the cells:

(2) $$\%\text{ of Total protein=}\left(\frac{\text{Total protein after pretreatment}}{\text{Total protein in cell}-\text{control}}\right)\times 100$$

### Gel Electrophoresis

The stability of the proteins after pre-treatment with IL was confirmed by native gel electrophoresis. The cells after pre-treatment were suspended in 50 mM phosphate buffer and disrupted by bead beating (see procedure in section Protein Content). The supernatant was diluted 1:1 with native sample buffer. The diluted sample (~25 μl) was then applied on 4–20% Criterion TGX, Tris glycine precast gel and run with 10 × Tris glycine native buffer at 125 V for 75 min. The gel was stained with Pierce™ Silver Stain Kit. The material for electrophoresis was bought from Bio-Rad and staining kit was purchased from Thermo Fisher Scientific.

### Carbohydrate Analysis

The total carbohydrate content of IL pre-treated cells was determined by acid hydrolysis, adapted from Dubois et al. (1951). The IL pre-treated cells were suspended in water such that the final cell concentration was 1 mg/ml and disrupted by bead milling (see procedure in section Protein Content). To 50 μl of this suspension, 450 μl water, 500 μl of 5% phenol solution, and 2.5 ml of concentrated sulfuric acid were added. The mixture was incubated at room temperature for 10 min and then at 35°C in a water bath for 30 min. The carbohydrates react with acidic phenol to give a yellow orange color that was then measured at 483 nm using a UV spectrophotometer (Beckman). For control process, the cells were directly suspended in water without any pre-treatment. Starch samples were measured as positive controls. All samples were measured in duplicate. The calibration curve was prepared using glucose as the standard. The total carbohydrate content is expressed as:

(3) $$\begin{array}{l} \%\text{ Total carbohydrate content}\\ \;=\left(\frac{\text{Total carbohydrate after pretreatment}}{\text{Total carbohydrate in the cell}-\text{control}}\right)\times 100 \end{array}$$

## Results

### Overview

Extraction efficiency of different components (e.g., proteins, carbohydrates, lipids) after IL pre-treatment is studied and the protein stability is determined by electrophoresis. The study discusses the impact of ILs on lipid extraction efficiency and impact of pre-treatment using ILs on separation of different components from microalgae.

### IL Lipid Extraction Efficiency

Preliminary screening of two IL classes, imidazolium-, and phosphonium-based ILs (see Table 1), on lipid extraction from intact freeze-dried *N. oleoabundans* shows in Table 2 the highest lipid extraction efficiency for TBP SO_4_ and BMIM DBP (experiments carried out in duplicate).

**Table 2:** Effect of different ILs on *N. oleoabundans* permeability (lipid extraction).

	Control^*^	**TBP SO_**4**_**	**TBP TOS**	**BMIM DBP**	**EMIM DBP**	**BMIM DCA**	**EA**
% of fatty acids/mg biomass	~25	~10	~2	~9	~1	~2	~0

* *Chloroform-MeOH extraction of lipids*.

**Figure 3:**
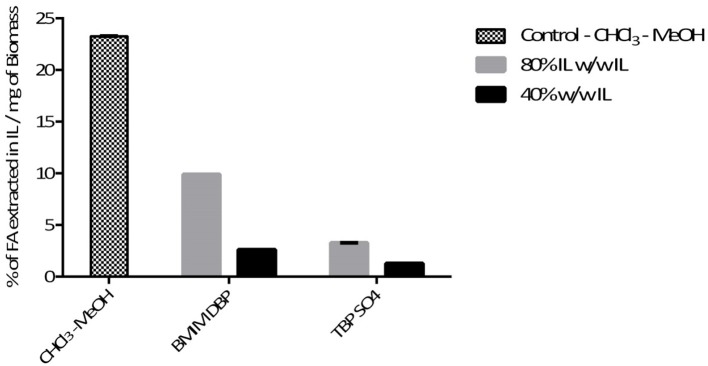
Effect of IL concentration on extraction of lipids at 45°C.

Further pre-treatment studies of freeze-dried *N. oleoabundans* with imidazolium (BMIM DBP)-based IL showed better lipid extraction efficiency compared to phosphonium (TBP SO_4_)-based ILs (see Figure 3) at concentrations of 40 and 80% w/w and at a temperature of 45°C. However, TBP SO_4_ could have an impact on the cell wall and was hence selected for further studies.

### IL Pre-treatment and Extraction of Microalgae Components

Microalgae biomass also contains a large amount of proteins and carbohydrates besides lipids. Additional studies were performed to recover these components in their native form after biomass pre-treatment with ILs.

**Figure 4:**
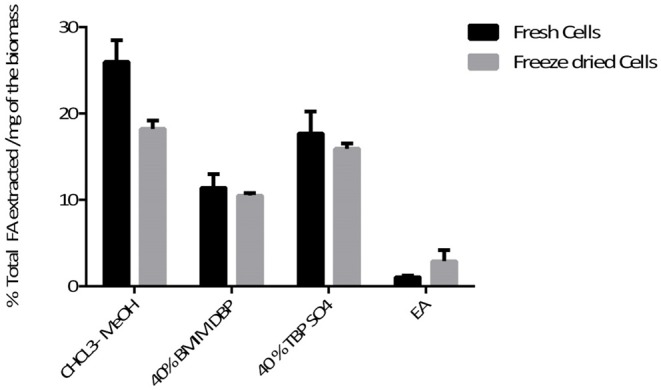
Total lipids extracted with ethyl acetate after IL pre-treatment at 45°C.

Pre-treatment studies were done on both fresh and freeze dried *N. oleoabundans* cells using 40% w/w solution of BMIM DBP and TBP SO_4_. Hydrophobic components were subsequently extracted with ethyl acetate and then the cells were mechanically disrupted to recover hydrophilic components. The amount of fatty acid extracted after pre-treatment was compared with the Bligh and Dyer (control using CHCl_3_-MeOH) method. The amount of lipid extracted from fresh biomass after pre-treatment was 11.4% and 17.7% per milligram of biomass for BMIM DBP and TBP SO_4_ compared to 26% per milligram of biomass using the Bligh and Dyer method. For the freeze-dried biomass, the amount of lipid extracted after pre-treatment was 10.5 and 15.9% per milligram of biomass for BMIM DBP and TBP SO_4_ compared to 18.2% per milligram of biomass using the Bligh and Dyer method. The results (see Figure 4) show that lipid recovery was better with TBP SO_**4**_ in comparison to BMIM DBP for both fresh and freeze-dried cells.

**Figure 5:**
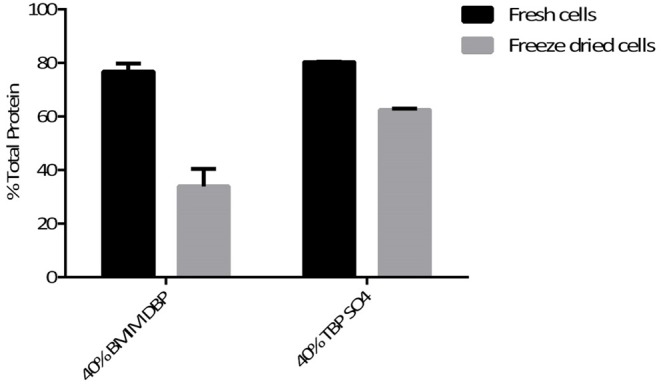
Total proteins in the biomass after IL pre-treatment at 45°C.

The hydrophilic components, proteins, and carbohydrates after lipid extraction were recovered by cell disruption. The percentage of total protein recovered after pre-treatment using BMIM DBP and TBP SO_4_ was 76.8 and 80.3% for fresh cells, and for freeze dried cells, 33.8 and 62.5%, respectively (see Figure 5), were observed.

**Figure 6:**
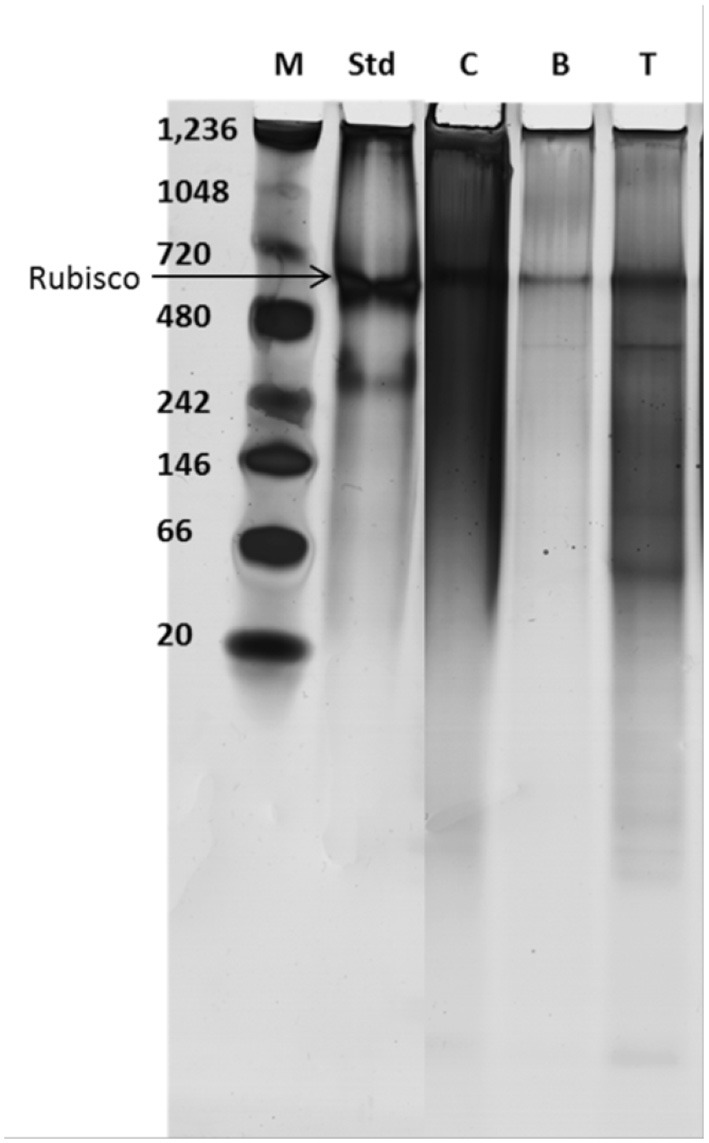
Protein stability determined by native gel electrophoresis; M, marker; Std, standard Rubisco; C, control supernatant after bead beating the cells; B and T, After treatment with BMIM DBP and TBP SO_4_, respectively, cells suspended in water and bead beated.

The proteins recovered after extraction of lipids were run on a native gel and detected using silver stain (see Figure 6) and the multi-component protein Rubisco indicated.

**Figure 7:**
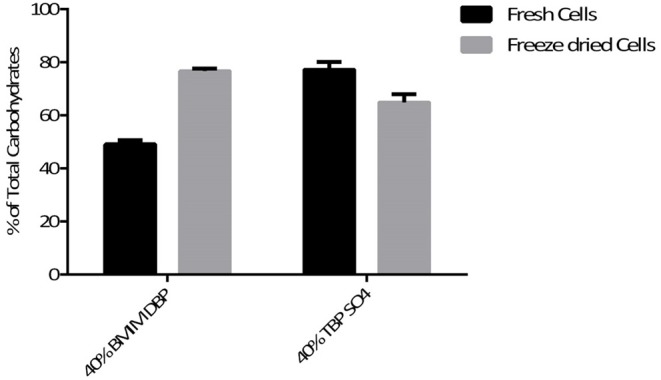
Total carbohydrates in biomass after IL pre-treatment at 45°C.

Additionally, the aqueous phase after cell disruption was analyzed for carbohydrate content. The percentage of total carbohydrate recovered after pre-treatment using BMIM DBP and TBP SO_4_ was 49 and 77.1% for fresh cells and 74.6 and 64.8%, respectively, for freeze-dried cells (see Figure 7).

A summary of the biomass components separated by IL pre-treatment is presented in Table 3. The results thus show that the microalgae components lipids, proteins, and carbohydrates can be selectively fractionated after IL pre-treatment and whereby the proteins retain their full functional composition.

**Table 3:** Summary of biomass components separated under different IL pre-treatment.

	**Fresh cells**			**Freeze-dried cells**		
	*Control	**40% BMIM DBP**	**40% TBP SO_**4**_**	*Control	**40% BMIM DBP**	**40% TBP SO_**4**_**
% of total fatty acid/mg of biomass	26.0	11.4	17.7	18.2	10.5	15.9
% of total fatty acid in cells	100	43.9	68.0	100.0	57.6	87.4
Proteins/mg of biomass	16.2	12.4	13.0	21.4	7.2	13.4
% of total protein in cells	100.0	76.8	80.3	100.0	33.8	62.5
Carbohydrates/mg of biomass	16.8	8.2	12.9	21.7	16.6	14.0
% of total carbohydrate in cells	100.0	49.0	77.1	100.0	76.6	64.8

a *Control—No pre-treatment with IL. Cells lysed by bead beating and analyzed for content as described in the Materials and Methods section*.

**Figure 8:**
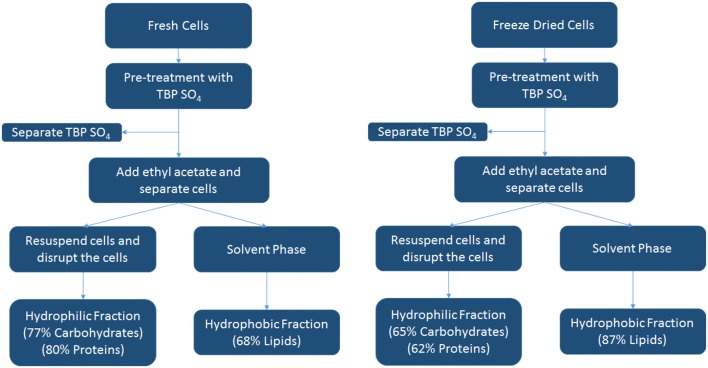
Overview of fractionation process with fresh and freeze-dried cells using the IL TBP SO_4_.

A schematic overview of the IL pre-treatment studies with TBP SO_4_ for both fresh and freeze-dried cells using the data of Table 3 is shown below in Figure 8. This scheme shows the different products as a hydrophilic/hydrophobic fraction.

## Discussion

### IL Lipid Extraction Efficiency

In this study, the extraction efficiencies of BMIM DBP (imidazolium)- and TBP SO_4_ (phosphonium)-based ILs were investigated after initial screening with different ILs (see Table 1). As the concentration of IL increases from 40 to 80% w/w at 45°C, the amount of lipid extracted increases from 2.61 to 9.89% per milligram of biomass for BMIM DBP and from 1.28 to 3.27% per milligram of biomass for TBP SO_4_ (see Figure 3)_._ This increase in extraction capacity could be attributed to the increase in hydrophobicity of the IL solution. IL solutions under mild conditions were able to extract lipids from intact microalgae cells; the maximum amount extracted was ~42% of the total fatty acid present in the cells (this value is calculated as % of total fatty acid in the cells, wherein the total fatty acid in cell is measured by Bligh and Dyer—control). Based on the results in Figure 3, BMIM DBP could permeabilize the cells and extract lipids better than TBP SO_4_, indicating that the cation and anion influences the extraction efficiency, but to a different degree. The results also confirm that the lipid solubility in aqueous solution of ILs is low. Although TBP SO_4_ shows low lipid extraction efficiency, it might still have an influence on the cell wall. The hypothesis is that the hydrogen bonding network of the cell wall is disrupted, leading to the formation of pores through which lipids can leak out. ILs are known to solubilize natural polymers such as cellulose and pectin by direct IL interaction. Hydrophilic ILs displaying low viscosities and high hydrogen bond capacity are reported to be more efficient in the solubilization process (Brandt et al., 2013; Lee et al., 2017). In other studies by different authors (Kim et al., 2012; Teixeira, 2012; Fujita et al., 2013; Choi et al., 2014; Olkiewicz et al., 2015) wherein the IL pre-treatment was done at temperatures close to 100°C, lipids released were extracted with organic solvent and extraction efficiency was >90% of the total fatty acid content. Thus, it indicates that temperature is indeed an important factor influencing the extraction efficiency.

### IL Pre-treatment and Extraction of Microalgae Components

The above studies showed that aqueous IL solutions could permeabilize the cells as well as extract the lipids without cell disruption. This observation is in accordance with our previously published studies (Desai et al., 2016) that aqueous IL solutions could permeabilize the intact microalgae cells under mild conditions and release the intracellular hydrophobic pigments (Desai et al., 2016).

Microalgae biomass also contains a large amount of proteins and carbohydrates besides lipids. Additional studies with fresh and freeze-dried cells were performed to recover these components in their native form after biomass pre-treatment with ILs. The results (see Figure 4) show that lipid recovery was better with TBP SO_4_ in comparison to BMIM DBP for both fresh and freeze-dried cells. This shows that TBP SO_4_, which has a low lipid extraction capacity, even at 40% w/w concentration, is able to permeabilize the cells and released lipids are subsequently extracted with ethyl acetate. The release of intracellular content with freeze-dried cells could be attributed partially to the drying effect of the cell walls, which makes it more permeable. The results also show that ethyl acetate alone is not able to permeabilize the cells and extract the lipids. Additionally, studies using hexane instead of ethyl acetate for extracting lipids after IL pre-treatment were performed (not shown). The results indicated that no lipids were extracted in the hexane phase, and this could prove a possible cooperative role of ethyl acetate together with IL in permeabilizing the cell wall so that lipids can be efficiently extracted.

The hydrophilic components, proteins, and carbohydrates after lipid extraction are recovered after cell disruption. The percentage of total protein recovered after pre-treatment using BMIM DBP and TBP SO_4_ was 76.8 and 80.3% for fresh cells and 33.8 and 62.5% for freeze-dried cells, respectively (see Figure 5). The decrease in protein recovery for freeze-dried cells could be due to direct contact of IL with proteins in the already compromised cell wall. In a separate study, aqueous solution of BMIM DBP (results not shown) and TBP SO_4_ (Desai et al., 2014) in contact with Rubisco (Ribulose-1,5-bisphosphate carboxylase/oxygenase) causes aggregation/precipitation of the protein molecule. These results thus indicate that TBP SO_4_ effectively permeabilizes the cell wall such that proteins remain intact inside the cell and can be recovered in their functional state after cell disruption. The proteins recovered after extraction of lipids were run on a native gel and detected using silver stain (see Figure 6). Although microalgae contains other proteins, Rubisco is used as the known biomarker protein for microalgae. The native gel shows that Rubisco remains intact and is not dissociated into its subunits, indicating that proteins recovered after IL pre-treatment retains its native form.

Additionally, the aqueous phase after cell disruption was analyzed for carbohydrate content. The percentage of total carbohydrate recovered after pre-treatment using BMIM DBP and TBP SO_4_ was 49 and 77.1% for fresh cells and 74.6 and 64.8%, respectively, for freeze-dried cells (see Figure 7).

A summary of the biomass components separated by IL pre-treatment is presented in Table 3, and for the IL TBP SO_4_, a schematic is also presented in Figure 8. The results thus show that the microalgae components lipids, proteins, and carbohydrates can be selectively fractionated after IL pre-treatment and whereby the proteins retain their full functional composition.

## Conclusion

In this article, pre-treatment of *N. oleoabundans* using ILs and subsequent fractionation into hydrophilic and hydrophobic components was studied for both fresh and freeze-dried biomass. Additionally, the lipid extraction efficiency of aqueous IL solution under different concentration conditions was studied. We have demonstrated that aqueous solution of imidazolium- and phosphonium-based ILs was able to extract lipids from intact microalgae, albeit to a different degree. We have also shown that pre-treatment of microalgae with BMIM DBP and TBP SO_4_ at low concentration (40% w/w) results in permeabilization of cells. The biomass can then be fractionated into hydrophilic and hydrophobic components whereby the proteins were recovered without losing their nativity. The recovery of total fatty acids was ~68% and that of proteins and carbohydrates was ~80 and 77%, respectively, of the total amount present in the cells, after pre-treatment of fresh biomass with TBP SO_4_. Most of the current processes that use energy-consuming mechanical cell disruption (e.g., bead milling, high-pressure homogenization) (Günerken et al., 2015) and solvents such as methanol/chloroform and hexane (Cuellar-Bermudez et al., 2015) are able to recover only lipids and render the proteins unsuitable for use due to denaturation/degradation. This article is a step forward in establishing the role of ILs in microalgae biorefinery by developing a novel selective fractionation concept for both hydrophobic compounds (e.g., lipids) and hydrophilic compounds (e.g., proteins, carbohydrates).

Pre-treatment studies as described in this article show the novelty of separating lipids without mechanical disruption and subsequent separation of hydrophilic components (proteins, carbohydrates) in their native form after cell disruption. The process can be optimized further to improve the yields. There are various parameters that influence the efficacy such as biomass loading, time of contact with IL and organic solvent, amount of solvent added, and type of IL, and these should be investigated in detail.

While ILs indeed have a potential role to play in microalgal biorefinery, it can only be realized if they are biocompatible, biodegradable, and economical. The ILs must be tested for their reusability and recyclability so as to make the process economically viable. To be able to judge a process superior than other would require a systematic approach and certain criteria, on basis of which the process is evaluated. Most of these studies are at their infancy and should be evaluated in terms of energy consumption, efficacy, and cost. In the past years, a few studies were published (Ruiz et al., 2016; Chia et al., 2018) about economic and environmental aspects of microalgae biorefinery for biofuel and also on high value product perspectives (Vanthoor-Koopmans et al., 2012; Chew et al., 2017).

## Data Availability Statement

The datasets generated for this study are available on request to the corresponding author.

## Author Contributions

RD and MF performed the experiments. ME and RW analyzed the data. RD wrote a first draft. All authors commented on the manuscript, which was finalized by ME.

### Conflict of Interest

The authors declare that the research was conducted in the absence of any commercial or financial relationships that could be construed as a potential conflict of interest.

## Abbreviations

IL: Ionic Liquid
TBP SO_4_: Tributylmethylphosphonium methylsulfate
BMIM DBP: 1-Butyl-3-methylimidazolium dibutylphosphate.
